# Henipavirus Mediated Membrane Fusion, Virus Entry and Targeted Therapeutics

**DOI:** 10.3390/v4020280

**Published:** 2012-02-13

**Authors:** Deborah L. Steffen, Kai Xu, Dimitar B. Nikolov, Christopher C. Broder

**Affiliations:** 1 Department of Microbiology and Immunology, Uniformed Services University, Bethesda, MD 20814, USA; Email: Deborah.Steffen@usuhs.mil; 2 Structural Biology Program, Memorial Sloan-Kettering Cancer Center, New York, NY 10021, USA; Email: xuk@mskcc.org (K.X.); nikolovd@mskcc.org (D.B.N.)

**Keywords:** paramyxovirus, entry, attachment glycoprotein, fusion glycoprotein, membrane fusion, receptor, ephrin-B2, ephrin-B3, inhibitor, antibody, immunotherapy

## Abstract

The *Paramyxoviridae* genus *Henipavirus* is presently represented by the type species Hendra and Nipah viruses which are both recently emerged zoonotic viral pathogens responsible for repeated outbreaks associated with high morbidity and mortality in Australia, Southeast Asia, India and Bangladesh. These enveloped viruses bind and enter host target cells through the coordinated activities of their attachment (G) and class I fusion (F) envelope glycoproteins. The henipavirus G glycoprotein interacts with host cellular B class ephrins, triggering conformational alterations in G that lead to the activation of the F glycoprotein, which facilitates the membrane fusion process. Using the recently published structures of HeV-G and NiV-G and other paramyxovirus glycoproteins, we review the features of the henipavirus envelope glycoproteins that appear essential for mediating the viral fusion process, including receptor binding, G-F interaction, F activation, with an emphasis on G and the mutations that disrupt viral infectivity. Finally, recent candidate therapeutics for henipavirus-mediated disease are summarized in light of their ability to inhibit HeV and NiV entry by targeting their G and F glycoproteins.

## 1. Introduction

Hendra virus (HeV) and Nipah virus (NiV) are enveloped, negative sense single-stranded RNA viruses that were first identified in the mid 1990s following two separate outbreaks in Australia and Malaysia, respectively [1,2,3]. Both outbreaks began as zoonotic respiratory infections in either horses (HeV) or swine (NiV) that were transmitted to humans in close contact with infected animals (reviewed in [4]). For both HeV and NiV the initial symptoms in infected humans manifest ~1 to 2 weeks post infection with fever, chills, headache and myalgia but can progress to severe respiratory distress and/or acute encephalitis [1,5,6,7,8,9,10]. Both NiV and HeV can also cause relapsed encephalitis in infected individuals several months to several years following recovery from acute infection [11,12,13]. In nature both HeV and NiV are found in several pteropid fruit bat species (flying foxes) [14], but the routes of transmission to humans are different as HeV is transmitted from bats-to-horses and then to humans, while NiV transmission has been from bats-to-pigs and then to humans and also directly from bats-to-humans as well as humans-to-humans [15,16,17,18] (reviewed in [19]). 

Only sporadic outbreaks of HeV occurred in the years immediately following its initial recognition in 1994, but starting in 2006 annual outbreaks of HeV have occurred among the equine population in Australia, resulting in a total of seven human cases with four fatalities [8,20]. However, in 2011 (June to October) an unprecedented number of eighteen independent outbreaks of HeV among horses in Australia was reported, culminating in the death or euthanasia of 23 horses, 1 dog and the close monitoring of more than 60 individuals for HeV infection [21,22]. Similarly, following the first outbreak in Malaysia and Singapore in 1998, almost annual outbreaks of NiV infection with high human case fatality rates (~70%) have been recorded primarily in Bangladesh but also India (reviewed in [23,24]). To date, there have been a total of 564 reported cases of NiV infection in people of which 299 have been fatal (reviewed in [23]). The repeated occurrences of henipavirus outbreaks among people and animals has demonstrated the importance for developing both intervention and countermeasure strategies to prevent cases of human infection [25,26,27]. 

While outbreaks of HeV and NiV have been contained to Australia (HeV) and Malaysia, Bangladesh and India (NiV), additional serological and limited nucleic acid evidence suggests that antigenically related henipaviruses are circulating in other regions including Thailand, Indonesia, China, Madagascar and West Africa [28,29,30,31,32,33,34]. Further serological evidence (cross-reactive antibodies to NiV glycoproteins) has also suggested that henipavirus transmission to domestic pigs in West Africa is possible [35]. In addition to the apparent widespread occurrence of the henipaviruses in various bat species, and unlike all other paramyxoviruses, HeV and NiV possess a broad host tropism that includes pigs, horses, cats, dogs, guinea pigs, hamsters, ferrets, monkeys and humans, leading to the classification of HeV and NiV into the new genus *henipavirus* in the family *Paramyxoviridae* [36]. Given the high morbidity and mortality rates associated with henipavirus infections in both humans and livestock, their recognized natural reservoirs in nature, the ease of propagation and a lack of any licensed vaccines or therapeutics, HeV and NiV pose significant biosecurity threats and are classified as biosafety level-4 (BSL-4) pathogens. 

Virus attachment, membrane fusion and particle entry for HeV and NiV requires two distinct membrane-anchored glycoproteins: an attachment glycoprotein (G) and a fusion glycoprotein (F). The G glycoprotein is required for receptor binding and virion attachment to the host cell, and the F glycoprotein is directly involved in facilitating the merger of the viral and host cell membranes. As HeV-G and -F share a high degree of similarity with NiV-G and -F (approximately 83% and 89% amino acid identity for G and F, respectively), it also seems reasonable that the characteristics and features attributed to the viral glycoproteins of one virus may be representative of the corresponding viral glycoproteins of the other virus [37].

## 2. Attachment Glycoprotein (G)

Most of the well-described paramyxoviruses possess a multifunctional hemagglutinin–neuraminidase (HN) glycoprotein which binds the virions to sialic acid receptors on host cells, whereas some others, such as the morbilliviruses including measles virus (MeV), have an H attachment glycoprotein, which possesses only hemagglutinating activity, and uses the membrane proteins CD46 or CD150/SLAM as receptors, depending on the virus strain (reviewed in [38,39]). Recently, the adherens junction membrane protein nectin-4 on human epithelial cells has also been shown to be an important receptor for MeV [40,41]. Like the HN and H glycoproteins, the henipavirus attachment G glycoprotein is a type II transmembrane protein that consists of an N-terminus cytoplasmic tail, a transmembrane domain, a stalk domain and a globular head; however the G glycoprotein possess neither hemagglutinin nor neuraminidase activities. The globular head folds as a β-propeller with a central cavity surrounded by six blades, which themselves are composed of four anti-parallel beta sheets [42,43,44]. The β-propeller shape is maintained by disulfide bonds between beta sheets in each blade as well as two additional disulfide bonds between blades three and four and between the N- and C-termini of the globular head. Five potential N-linked glycosylation sites (N306, N378, N417, N481 and N529) have been identified in the globular head of NiV, and evidence has verified that four of the five sites are glycosylated with one site, N417, yielding variable reports likely owing to alternative expression methods [43,44,45]. Likewise, the HeV-G head domain also has the same five predicted and conserved N-linked glycosylation sites occupied by carbohydrate moieties [46]. Detailed glycan composition and site occupancy analysis of the entire ectodomain of HeV-G has recently been performed and has also revealed O-linked glycosylation sites in the protein [47].

### 2.1. Oligomerization of G Glycoprotein

**Figure 1:**
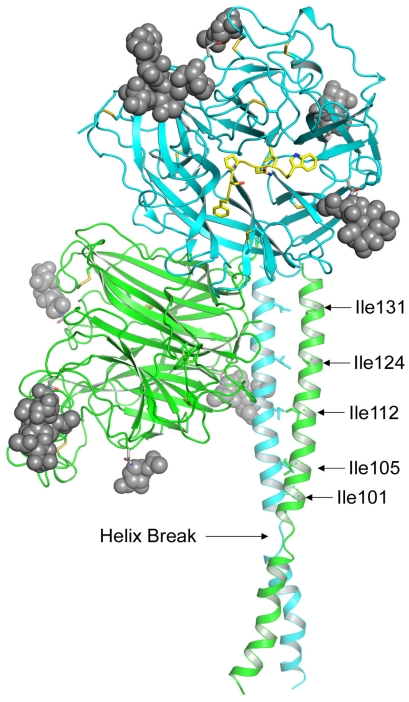
Model of the Hendra virus attachment G glycoprotein. The HeV-G ectodomain is shown in its dimer conformation. The secondary structure elements of the two globular head domains, colored in green and blue, are derived from the crystal structure, which also revealed the five predicted N-linked glycosylation sites (N306, N378, N417, N481 and N529) occupied by carbohydrate moieties (gray spheres) [46,47]. However, N378 was not modeled in the figure due to weak electron density. The G glycoprotein head domain folds as a six-blade β-propeller with disulfide bonds illustrated as yellow sticks. The residues of the ephrin-B2 G-H loop are shown in yellow. While the entire structure of the HeV-G stalk domain (residues 71–173) has not been determined, residues 77–136 are modeled for each monomer suggesting this region forms a discontinuous helix (Helix Break) [50]. The position of the HeV-G head dimer and stalks are oriented based on the alignment with the NDV structure and the receptor binding face of the blue monomer is facing out and the green monomer is facing left. Despite having two helical ranges, Thr-77 to Lys-95 and Thr-98 to Ser-135, the HeV-G stalk residues 98–135 appear equivalent to the HN glycoprotein stalk helix domain of the recently reported NDV structure [49]. Additionally, the Ile residues in the HeV-G stalk domain that can modulate conformational changes associated with receptor binding are indicated and are located in the alpha helical region of the HeV-G stalk domain that aligns with the NDV-HN stalk [51].

The native conformation of G when expressed on the virion or the surface of an infected cell is a tetramer, which is comprised of a dimer of dimers [44,48]. Residues responsible for the oligomerization of G are isolated to the stalk domain as expression of the globular head alone results only in monomeric species [44]. Further investigation determined that two disulfide bonds in the stalk domain of G enable dimer formation, but the specific interactions in the stalk domains between homodimers that enable G to form a tetramer are unknown [48]. Bowden *et al.* proposed that one surface of dimer-dimer interface occurs across the β1- and β6-propellers of the globular head [44,45]. This suggestion is supported by the lack of both structural divergence and N-glycosylation sites, which would interfere with oligomerization, along this section of the protein. Additionally, the recently reported structure and model of a tetrameric Newcastle disease virus (NDV) HN has provided further insight on the organization and oligomeric structure of a paramyxovirus attachment glycoprotein. The stalk domains of NDV-HN form a four-helix bundle (4 HB) with a hydrophobic core that is the result of an 11-residue repeat domain in the stalk [49]. Similarly to NDV-HN, HeV and NiV-G stalks contain alpha helices with a predicted break from amino acids 95–98, and the modeled juxtaposition of these stalks with the globular heads of HeV-G resembles the tetrameric structure of NDV-HN (Figure 1). 

A model of a tetrameric parainfluenza 5 virus (SV5) HN previously reported suggested a tetramer formation in which the globular heads of the two dimers were in contact [52], which contrasts with the more recent structural data of the NDV tetramer in which the globular heads of the dimers are separated [49]. Although the globular head dimer of NDV and SV5 can be superimposed with a low 1.5 Å root mean square deviation (rmsd), the earlier SV5 tetramer model is not in accord with the recent NDV dimer and stalk configurations. Given the characteristics of HeV and NiV-G, specifically the location of the N-linked glycosylation sites and the predicted stalk helices, it seems reasonable that the tetrameric form of the henipavirus G glycoprotein would also resemble the NDV-HN structural model (Figure 1). 

## 3. Receptors Ephrin-B2 and Ephrin-B3

The henipaviruses are the most recently recognized paramyxoviruses that also use host cell membrane proteins as virus entry receptors, and both HeV and NiV bind to ephrin-B2 and ephrin-B3 via their G glycoproteins [53,54,55,56]. Human ephrin-B2 and ephrin-B3 are 39% identical in amino acid sequence and are members of a large family of surface expressed glycoprotein ligands that bind to Eph receptor tyrosine kinases and mediate bi-directional cell-cell signaling events within the nervous, skeletal and vascular systems [57,58]. The ephrin-B2 and -B3 molecules are highly conserved proteins across species with amino acid identities ranging from 95–96% and 95–98%, respectively, including those hosts susceptible to henipavirus infection such as human, horse, pig, cat, dog and flying foxes [59]. 

Ephrin-B2 is found in arteries, arterioles and capillaries in multiple organs and tissues including arterial smooth muscle and human bronchiolar epithelial cells but appears absent from venous components of the vasculature [60], whereas ephrin-B3 is found predominantly in the nervous system and the vasculature (reviewed in [61,62]). The identification of ephrin-B2 and ephrin-B3 as functional receptors for the henipaviruses in cultured cells provides some explanation for both the broad species tropisms of the viruses, owing to their highly conserved nature, and the observed distribution of viral antigen in arterial endothelial cells, smooth muscle, neurons, and some epithelial cells (reviewed in [63,64]). 

While it is unclear how many ephrin molecules are required to bind oligomeric G to activate the henipavirus membrane fusion process, recent structural data has revealed that a G glycoprotein head domain (monomeric) binds an ephrin molecule in a 1:1 ratio [42,43,46]. Both ephrin-B2 and ephrin-B3 are able to support productive infection of HeV and NiV, but the binding affinities of HeV and NiV-G for ephrin-B2 and -B3 are uncertain and this is also complicated by the oligomeric nature of both the G glycoprotein and the ephrins. One report has suggested HeV and NiV-G have the same binding affinity for ephrin-B3 while NiV-G has a higher affinity for ephrin-B2 than HeV-G; however another study indicated that HeV and NiV-G bound ephrin-B2 similarly while NiV-G engaged ephrin-B3 with a higher affinity in comparison to HeV-G [59,65,66]. One possible explanation to explain these different findings is that two different HeV-G sequences were used. Negrete *et al.* determined that the sequences of two HeV-G strains currently used in research contain one amino acid change at position 507, having either a Ser or a Thr [66]. A Thr at position 507 confers ephrin-B3 affinity similar to that of NiV-G, but a Ser at position 507 reduces the affinity of HeV-G for ephrin-B3 while having no affect on ephrin-B2 affinity. Given the physiological locations of ephrin-B2 and -B3, the observed differences in the transmissibility of HeV and NiV and the differences in HeV and NiV disease course in susceptible hosts upon infection, additional study of the henipavirus G glycoproteins and their interaction with the ephrin receptors will further our understanding of the biology and pathology of these important zoonotic agents. 

### 3.1. Interaction of Henipavirus G with Ephrin Receptors

B-class ephrins contain a globular domain that is comprised of eight β-strands (identified as A-D, F-H and K) surrounding a hydrophobic core [67]. Although there are three B-class ephrins (B1-B3) with high levels of similarity, only ephrin-B2 and ephrin-B3 are able to serve as functional receptors for HeV and NiV. The major structural divergence between these ephrins occurs in the G-H loop, which is a 15 amino acid linker region between β-strands G and H that is also primarily responsible for the binding between ephrins and their cognate Eph receptors [68]. 

Despite the 15-amino acid length of the G-H loop, only a short stretch of conserved amino acids (F/Y_117/120_SPNLW_122/125_) binds in the groove of the globular head of HeV and NiV-G with the only difference between ephrin-B2 and ephrin-B3 being F117 and Y120, respectively [42,43,68]. (To avoid confusion, we will use the ephrin-B3 numerical designation for the G-H loop residues, identified as F/Y_120_-W_125_). Preceding the solution structures of the henipavirus G glycoproteins in complex with ephrin-B2 and -B3, the importance of the ephrin G-H loop was first hypothesized by Negrete *et al.* and confirmed by mutagenesis that involved the conversion of two residues in the G-H loop of ephrin-B1 to the residues in ephrin-B2, making ephrin-B1 a functional NiV receptor [55]. The ephrin-B1 amino acid replacements L124→Y and W125→M would result in steric clashes and converting these residues to match the ephrin-B3 sequence eliminates this hindrance [55]. The overall conformation and flexibility of the ephrin-B2 and ephrin-B3 G-H loops might also influence receptor selectivity of the henipavirus G glycoproteins. Indeed, both ephrin-B2 and -B3 appear to have G-H loops with extended and relatively rigid conformations, whereas ephrin-B1 has a more flexible G-H loop, which may not be compatible with the apparent lock-and-key ephrin/G glycoprotein binding mechanism [69].

Binding between ephrin-B2 and HeV and NiV-G occurs in two regions that occlude approximately 2,700 Ǻ^2^ surface area on the interface [42,43]. The first region occurs along a mostly polar area of the outer rim of the cavity in the globular head of the henipavirus G and requires 24 hydrogen bonds, four salt bridges and multiple hydrophobic interactions [42,43]. The second region primarily involves interaction through van der Waals forces of the ephrin G-H loop residues F/Y120, P122, L124 and W125 with binding pockets in the central cavity of the globular head of G [42,43]. The four binding pockets in HeV and NiV-G for the residues in the ephrin-B2 and -B3 G-H loop are highly conserved with only four differences, the most notable being a Val (NiV) to Thr (HeV) change at position 507 [42] (Table 1). 

The crystal structures of both HeV and NiV-G in complex with ephrin-B2 have provided much information on the specific interaction between these attachment glycoproteins and receptor, but whether relevant conformational changes must occur for G/ephrin-B2 binding is not completely clear. It is generally accepted that the G-H loop of ephrin-B2 and -B3 does not undergo major conformational change, except for rearrangements of W125 upon G engagement that are suggested to “latch” ephrin-B2 into a stronger association [46]. 

**Table 1:** Henipavirus G glycoprotein receptor binding pockets. The residues in HeV-G and NiV-G that form the binding pockets for the residues of the ephrin-B2 and ephrin-B3 G-H loop are shown. The residues are highly conserved with those in bold-face indicating the four amino acids that are different between the HeV-G and NiV-G binding pockets for Eprhin-B2 and -B3 P122, L124 and W125.

Binding Pocket for loop residues	HeV-G glycoprotein residues	NiV-G glycoprotein residues
F/Y	C240, N557, A558, Q559, E579, I580, Y581, I588, R589	C240, N557, A558, Q559, E579, I580, Y581, I588, R589
P	P488, G489, Q490, E505, G506, **T507**, Q530, T531, A532	P488, G489, Q490, E505, G506, **V507**, Q530, T531, A532
L	**Y458**, W504, E505, G506	**F458**, W504, E505, G506
W	L305, **V401**, **N402**, W504	L305, **I401**, **R402**, W504

While there is debate as to whether major or minor conformational changes occur in the monomeric henipavirus G head domain upon receptor binding, it is clear that some limited structural rearrangements do occur but appear restricted to a specific region [42,43]. Three of the binding pockets in the HeV and NiV-G head domain (pockets for ephrin-B2 and -B3 residues P122, L124 and W125) undergo little conformational change, while the G glycoprotein D579-P590 loop (binding pocket for ephrin-B2 and -B3 F117 and Y120, respectively) appears to have the greatest structural alteration upon receptor binding [44]. Interestingly, this region is the one binding pocket that must accommodate two different residues, suggesting ephrin-B2 and -B3 bind to HeV and NiV-G by an induced-fit model as the F residue binding pockets in each henipavirus G glycoprotein must conform in a way to accommodate the different residues in the G-H loops of ephrin-B2 and ephrin-B3 [44,45]. Other more recent data suggest that ephrin-B2 binding to HeV-G supports a “lock, key and latch” model for the association between the G glycoprotein and its receptors with the W122 residue of ephrin-B2 serving as the “latch” [46]. Finally, it should also be recognized that the available structural data of a henipavirus G glycoprotein complexed with an ephrin receptor is from monomeric proteins and do not take into account the possibility of broader oligomeric changes that may occur following receptor engagement. For example, in case of MeV-H, it was recently reported that following receptor binding, the two heads of a MeV-H dimer move in relation to each other, and by stabilizing the H-dimer interface by inter-molecular disulfide bonds, fusion can be blocked, suggesting that oligomeric H conformational changes (dimer separation or movement) is linked to fusion triggering [70]. 

### 3.2. Mutations in the G Glycoprotein That Impact Function

**Figure 2:**
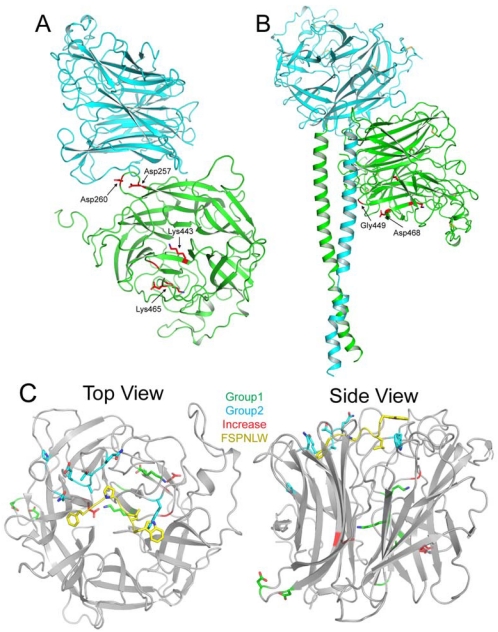
Mutations in HeV-G that affect fusion promoting activity. (**A**) Residues A257, A260, A443 and A465 that decrease HeV fusion when mutated are highlighted in red in the HeV-G globular head dimer. Based on the location of residues A257 and A260, it is likely they are necessary for interaction between the two globular heads, while residues A443 and A465 are centrally located in the globular head in a region of extensive hydrogen bonding. (**B**) The predicted structure of an HeV-G dimer with globular heads and stalk domains is shown with residues G449 and D468 highlighted in red. These two residues are located in proximity to the stalk domain, and mutation of these residues decreases HeV fusion, suggesting they may be involved in interactions between the globular heads and stalk domains that are essential for the fusion process. (**C**) Monomeric units of the globular head of HeV-G (gray) are shown in complex with the G-H loop residues FSPNLW of ephrin-B2 (yellow). Mutant Group 1 residues that decrease fusion are located further from the ephrin-B2 binding site and are shown in green. Mutant Group 2 residues that decrease fusion are shown in blue and cluster around the ephrinB2 binding site. Residues that enhance fusion (Group e1) are shown in red and are also distally located from the ephrin-B2 binding site. Given the divergent locations of the mutations that affect fusion, these mutations likely use different mechanisms, such as disrupting HeV-G structure or preventing ephrin-B2/B3 binding, to prevent HeV fusion.

As might be expected, mutational studies have highlighted a number of residues in both the HeV and NiV-G glycoproteins and the ephrin-B2 and -B3 receptors that are critical for their interaction. The majority of reported mutations in the G glycoprotein that affect function (*i.e.*, the protein’s fusion promoting activity in the context of co-expressed F glycoprotein) are conserved between HeV and NiV and are located throughout the globular head and stalk domains. Mutations located in the globular head that decreased fusion activation by at least 25%–50% can be separated into two groups based on their location in the globular head—distal or proximal to the ephrin binding site. Group 1 residues (D257A, D260A, K443A, G449A, K465A and D468A) are located closer to the stalk domain of the henipavirus G glycoprotein, and are distant from the ephrin binding site, but despite this lack of proximity, all of these Group 1 mutants display reduced binding to both ephrin-B2 and -B3 [56]. Given the distance between the locations of these mutations and the receptor binding site, it is unlikely that the mechanism of reduced binding is due to steric hindrance/blocking of the binding site. Bishop *et al.* proposed that these mutations could act by locking HeV-G in a conformation that favors binding to HeV-F but not ephrin-B2 as these mutants demonstrated an increase in HeV-F co-association [56]. A re-examination of some of these mutations in the context of the crystal structure of the dimeric interface of HeV-G provides some additional insight (Figure 2). As can be seen from the figure, mutants D257A and D260A appear to be involved in interaction between the two globular heads of the dimer, and disruption of the homodimer could lead to decreased receptor binding and resultant fusion activation (Figure 2A). Additionally, using the data reported by Yuan *et al.* on the NDV-HN tetrameric structure [49], modeling the location of the HeV-G stalk domain to examine the impact of the Group 1 mutants reveals that residues G449 and D468 appear to interact with the stalk domain. Some conformational changes in G occur upon receptor binding, and therefore HeV-G would have pre-receptor-bound and post-receptor-bound forms with structural differences in either the head or stalk (or both) domains. The importance of the HeV-G stalk domain in the context of its fusion promoting activity and role in the maintenance of the protein’s conformation is discussed below (Section 5). Mutation of either G449 or D468 could destabilize the pre-receptor-bound structure of HeV-G and interfere with receptor binding and/or receptor-triggered conformational changes required for its fusion promoting activity (Figure 2B). The final two mutants in Group 1 (K443A and K465A) are both located in a region of HeV-G that is part of a massive hydrogen bond network, which adds stability to the structure of HeV-G (Figure 2A). Mutation of these residues likely destabilizes this hydrogen bond network, preventing HeV-G from obtaining the correct conformation required for receptor binding and fusion. See Table 2 for a summary of mutant Groups.

The Group 2 residues (N402A, Q490A, E501A, W504A, E505A, Q530A, T531A, A532K, E533Q, N557A, Y581A and I588A) are located on the outer surface of the globular head near the ephrin binding site and can be further broken down into 3 sub-groups based on receptor usage in the context of either HeV or NiV-G [46,66,71] (Figure 2C and Table 2). The first sub-group (Group 2a) includes those residues that decreased fusion promoting activity by decreased receptor binding without knowing which ephrin receptor was used, and these mutations are Q530A, T531A, A532K and N557A [71]. The second and third sub-groups (Subgroups 2b and 2c) contain mutations that decrease the fusion promoting activity of G upon either ephrin-B2 usage (N402A, E501A, E505A, G506A, E533A, Y581A and I588A) or ephrin-B3 usage (N402A, Q490A, E501A, W504A, E505A, G506A, E533A, Y581A and I588A) respectively [46]. These two sub-groups of mutated residues are quite similar with only the additional Q490A and W504A mutations in Subgroup 2c that exhibit decreased fusion promoting activity in the context of HeV-G and ephrin-B3 interaction. Interestingly, the residues of Subgroup 2b had no apparent decrease in ephrin-B2 binding but were significantly impaired in supporting fusion promoting activity as measured by pseudotyped virus entry, whereas the majority of mutations in Subgroups 2a and 2c had decreased receptor binding. These findings were in good agreement with earlier observations that mutation of residues W504 and E505 impair ephrin-B3 binding and virus entry and mutation of E533 impairs both ephrin-B2 and -B3 usage [66]. In the context of HeV-G mutations, E505A also revealed impaired ephrin-B2 usage by virus entry but little effect on impaired binding, and mutation E533A, which abrogated both ephrin-B2 and -B3 usage in virus entry, had little effect on binding ephrin-B2 [46]. Only two mutations in Subgroup 2c (N402A and I588A) possess significant impairment of HeV-G function but have no decrease in ephrin-B3 receptor binding. Interestingly, among the Group 2 mutants, only two of these residues (E501 and E533) are not directly involved in forming binding pockets for the G-H loop of ephrin-B2 or -B3.

**Table 2:** Summary of HeV-G Mutants that affect fusion. Mutations that either decrease or increase HeV-mediated fusion are separated into various groups based on their location in the globular head (distal or proximal to ephrin-binding site) or stalk domain of HeV-G, which receptor is bound and whether the mutation affects receptor binding. Information supplied in the Notes column is based on the modeled structures of HeV-G head and stalk domains or obtained from experimental data as indicated. Squares shaded in gray represent information that is unknown or not relevant.

Effect	Location	Group	Subgroup	Residues	Binds	Notes
**Decrease**	Globular Head—Distal	Group 1		D257, D260		Disrupts dimer ^1^
				G449, D468		Disrupts stalk-head interaction ^1^
				K443, K465		Disrupts hydrogen bonding ^1^
	Globular Head—Proximal	Group 2	Subgroup 2a	Q530, T531, A532, N557	Unknown	Decreased receptor binding ^2^
			Subgroup 2b	N402, E501, E505, G506, E533, Y581, I588	Ephrin-B2	No disruption in receptor binding ^2^
			Subgroup 2c	Q490, E501, W504, E505, G506, E533, Y581	Ephrin-B3	Decreased receptor binding ^2^
				N402, I588		No disruption in receptor binding ^2^
	Stalk	Group 3		I101, I105, I112, I120, I124, I131, I138, I155, I160		No disruption in receptor binding ^2^
**Increase**	Globular Head	Group e1	Subgroup e1	D564, D470	Unknown	No disruption in receptor binding ^2^
				G439		Decreased receptor binding ^2^
			Subgroup e2	V401, Q490, Q559	Ephrin-B2	No disruption in receptor binding ^2^
				W504		Decreased receptor binding ^2^
			Subgroup e3	V401, Q559	Ephrin-B3	No disruption in receptor binding ^2^

^1^ Predictions based on modeled structure of HeV-G head and stalk domains; ^2^ Experimental data.

Group 3 mutations include a series of isoleucine residues in the stalk region of HeV-G (I101A, I105A, I112A, I120A, I124A, I131A, I138A, I155A and I160A) that also resulted in significant decreases in fusion promoting activity while having no effect on receptor binding, leading Bishop *et al.* to speculate that these residues are important for maintaining the conformational integrity and stability of the G glycoprotein as it relates to regulating the G-F interaction and its fusion promoting activity [51]. The region of the stalk domain that contains these residues is shown in Figure 1, and the implications of these mutations will be discussed below. 

Although the majority of mutations introduced in HeV and NiV-G resulted in decreased levels of fusion promoting activity, there were a few residues located in the globular head that enhanced fusion promoting activity upon mutation (Group e1) (Table 2). Again, these mutations can be divided into three groups based on their use of specific ephrin receptors. The first fusion-enhancing (e) group (Subgroup e1) of mutants (G439A, D564A and D470A) increased fusion promoting activity, although it was unknown which receptor was used for infection [56]. Interestingly, mutant G439A exhibited increased fusion promoting activity along with decreased receptor binding capacity. Fusion-enhancing Subgroups e2 and e3 contain mutants that increased fusion with ephrin-B2 (V401A, Q490A, W504A and Q559A) or ephrin-B3 (V401A and Q559A), respectively [46]. As might be expected the vast majority of these mutations in Subgroups e2 and e3 had no effect on receptor binding with the only exception being residue W504A in Subgroup e2 that had decreased receptor binding. 

Comparing some of the mutations using pseudotyped virus entry it was discovered that two mutations had opposite effects depending on whether the ephrin-B2 or ephrin-B3 receptor was utilized. Mutations Q490A and W504A both decrease the fusion promoting activity of G in ephrin-B3 dependent virus entry but also revealed enhanced virus entry in the context of ephrin-B2. Q490A is able to bind ephrin-B2 with an enhanced pseudotyped virus entry result but was unable to efficiently bind ephrin-B3, which resulted in a significant impairment of virus entry [46]. Further, HeV-G mutant W504A was notably impaired in binding both ephrin-B2 and ephrin-B3 in comparison to wild-type HeV-G; however this mutant has enhanced virus entry with ephrin-B2 expressing target cells. 

Finally, the importance of the G-H loop in ephrin-B2 and -B3 was first identified by Negrete *et al.* when the Leu and Trp residues in this solvent exposed loop were found critical for NiV binding and entry (described above) [55]. These authors also followed up these findings with NiV-G mutagenesis and identified several key residues in the receptor binding region of G that are critical for ephrin-B2 and/or ephrin-B3 binding [66]. The structures of the ephrin-B2 and -B3 complexes with henipavirus G glycoproteins are now known with the critical residues in the G-H loop indentified (F/Y_117/120_SPNLW_122/125_), and these residues have also been explored by mutagenesis in the context of ephrin-B2 [72]. Surprisingly, mutating four of these residues (S121, P122, L124 and W125) to an alanine increased the infectivity of NiV-F and -G pseudotyped virus, while F120A was the only ephrin-B2 mutant that decreased NiV pseudotyped virus infectivity [72]. Additional studies revealed that the enhanced infectivity of L124A was due to an increase in the binding between the ephrin-B2 L124A mutant and virus in comparison to wild-type ephrin-B2, while the increased infectivity of the other mutations where thought to be due to an increased plasticity in the G-H loop or the loss of steric hindrance in the binding pockets of NiV-G [72]. No single mutation could completely abrogate NiV pseudotyped virus infectivity suggesting that multiple residues in ephrin-B2 are important for interaction with NiV-G. 

Taken together, these mutations in both HeV and NiV-G and the ephrin receptor reveal an interesting perspective of the conformational changes that may be required for henipavirus envelope glycoprotein-mediated membrane fusion. Based on the predictive modeling of the HeV-G globular head dimer with the stalk domains (Figure 1), it seems clear that prior to receptor binding, HeV-G must be in a required pre-receptor-bound conformation, and disruption of this conformation, whether through disturbance of the dimer interface or the interaction of the globular head with the stalk, could have deleterious effects on receptor binding and the fusion promoting activity of G. Furthermore, it is also clear that ephrin-B2 and -B3 binding alone does not appear to be sufficient for triggering fusion because a number of HeV-G mutants are fully capable of binding ephrins but are completely blocked in promoting fusion. All together these observations suggest the existence of intermediate steps, initiated by receptor binding, that alter the conformation of G required for its fusion promoting activity. For example, the HeV-G mutant I588A is able to bind to both ephrin-B2 and -B3 to an equal if not better extent than wild-type HeV-G, yet it is unable to support pseudotyped virus entry, suggesting that important residues can be identified that appear to uncouple host cell/ephrin attachment and viral fusion initiation [46]. The observation that receptor binding and F triggering can be separated has also been made earlier with the MeV-H and -F glycoproteins [73,74]. This uncoupling of receptor binding and fusion initiation is further supported by the mutations in the HeV-G stalk domain, which also have no effect on receptor binding but are completely abrogated in supporting fusion [51]. These findings and the G glycoprotein mutants will be important tools for additional functional and structural analysis of the precise molecular steps underlying the receptor triggering mechanism of henipavirus entry and how such mutations in the G glycoprotein accomplish this functional block.

## 4. Fusion Glycoprotein (F)

While the G glycoprotein is required for attachment of the henipavirus virion to the target cell, the F glycoprotein is responsible for the merger of the viral membrane envelope with the target cell plasma membrane. The F glycoprotein is initially expressed as a 546 amino acid precursor (F_0_) which forms an oligomeric trimer that is cleaved into two subunits (F_1_ and F_2_) by the endosomal protease cathepsin L [75]. Unique to the henipaviruses, the processing of F_0_ into its biologically active form is a multi-step process requiring recycling of F_0_ from the cell surface into an endosomal compartment, mediated by an enodcytosis motif present in the cytoplasmic tail of F [76,77]. Interestingly, HeV and NiV-F glycoproteins contain no specific cleavage sequence, and cleavage is only inhibited by the deletion of six residues upstream of the cleavage site [78]. After cleavage, the homotrimer of disulfide bond linked F_1_ and F_2 _subunits is trafficked back to the cell surface.

The F_1_ subunit contains several important structural characteristics that include an N-terminal hydrophobic fusion peptide domain, two heptad repeat (HR) domains, a transmembrane domain and cytoplasmic tail. The two α-helical heptad repeat domains reside immediately downstream of the fusion peptide (HR1 or HRA) and upstream (HR2 or HRB) of the transmembrane domain and are the shortest HR domains among paramyxoviruses [79]. The C-terminal cytoplasmic tail and the transmembrane domain have also recently been implicated in modulating virus-mediated fusion as tyrosine residues in the tail have been shown to increase fusion activity and aid in the proper trafficking of F in polarized epithelial cells [80,81].

### 4.1. The Fusion Mechanism

The crystal structures of the paramyxovirus F glycoproteins (SV5 and human parainfluenza virus 3 (hPIV3)) have provided significant insight into the mechanism of the fusion process and the structural transition that occurs between the pre- and post-fusion conformations of F [82,83]. Although the paramyxovirus F glycoproteins resemble other Class I viral fusion glycoproteins, they are distinctly structurally different in comparison to other pre- and post-fusion Class I viral fusion glycoprotein structures, and the hPIV3-F and PIV5-F structures are the only available models for making good comparisons of the paramyxovirus F structural transition [84]. Prior to receptor binding, the hydrophobic fusion peptide located at the N-terminus of the F_1_ subunit is concealed in the protein. Upon receptor binding, the paramyxovirus attachment glycoprotein promotes F fusion activity by an as yet ill-defined mechanism, triggering irreversible conformational changes in F that (1) expose the fusion peptide, allowing it to be inserted into the opposing target cell membrane and (2) rearrange the HR domains, leading to the formation of the hallmark feature of Class I fusion—the six helix bundle (6 HB) (recently reviewed [39,85]). This process is a multi-step event that results in the elongation of F as well as the merger of the viral and cellular membranes. The assembly of the 6 HB is believed to provide the energy required for the membrane destabilization and merger event as the three HR2 domains within the F trimer are rearranged to bind via hydrophobic interactions in the grooves of the trimeric core composed of the HR1 domains [79]. However, the number of F homotrimers required for fusion pore formation and membrane merger is unknown. 

## 5. G and F Glycoprotein Interaction

Although recombinant forms of the paramyxovirus respiratory syncytial virus (RSV) and the F glycoprotein of SV5 are capable of mediating membrane fusion activity in the absence of their co-expressed G glycoprotein partner, the majority of paramyxoviruses, including the henipaviruses, require their attachment glycoprotein to promote F-mediated membrane fusion [86,87,88]. Despite the requirement of receptor engagement by henipavirus G to initiate fusion, the interaction of G and F appears independent of receptor binding as G and F can be co-precipitated in the absence of receptor [48,56,89,90]. It has been shown that several paramyxovirus F and attachment glycoprotein pairs, including those from MeV, NDV and hPIV2, first interact in the endoplasmic reticulum (ER) [91,92,93,94]. However, for other viruses (SV5 and hPIV3) an F and HN interaction prior to fusion was earlier suggested to not be robust [95], and here these viral F glycoprotein fusion systems do not suggest a model whereby the F and attachment glycoproteins are pre-associated (discussed in Section 6). Further, the henipavirus G and F glycoproteins have different and more complex trafficking patterns in comparison to other paramyxoviruses. The HeV and NiV-G have been shown to take longer than their partner F glycoprotein to traffic through the ER and Golgi, and this longer trafficking time of G and the complex pattern of F maturation suggests that G-F interaction does not occur until both glycoproteins are expressed on the cell membrane [91,96]. 

Although most evidence indicates that the G and F glycoproteins interact prior to G-ephrin receptor binding, the exact nature of this interaction and the domain(s) of G and F that associate are not well defined. Bishop *et al.* found that mutations at specific sites in the stalk domain of HeV-G inhibited HeV fusion due to an apparent loss of interaction with F [51]. These particular isoleucine residues are located in an alpha helical domain that resembles a heptad repeat that is highly conserved among paramyxoviruses [51]. Interestingly, the nine Ile→Ala mutations that abolished the fusion promoting activity of HeV-G are located near the region that Yuan *et al.* have implicated as important for the tetramer formation of NDV HN (Figure 1 and discussed above) [49,51]. Monoclonal antibody (mAb) binding analysis, with several mAbs that preferentially recognize G in complex with ephrin receptor, revealed that these HeV-G stalk domain mutants appeared to adopt a receptor-bound conformation in the absence of receptor binding and thus were unable to trigger F fusion activation even upon any subsequent ephrin receptor binding. These observations suggest that G must be in some correct pre-receptor bound tetrameric conformation in order to properly trigger F fusion activity and also indicate that receptor binding and fusion triggering by G can be uncoupled. 

## 6. Model of Henipavirus Fusion

Two principal models of paramyxovirus glycoprotein-mediated membrane fusion have been postulated [97] (recently reviewed [85]). In the first model, the F glycoprotein and the attachment glycoprotein are not physically associated in the membrane, but following receptor engagement there is an alteration in the attachment protein which facilitates its association with F and in so doing imparts or triggers/induces the F glycoprotein conformational changes leading to membrane fusion. This association or provocateur scenario has been supported by extensive functional and structural studies on the HN and F glycoproteins of hPIV3, NDV and PIV5 [49,95,98,99,100]. In the second model, the dissociation or clamp model, the F and attachment glycoproteins are pre-associated and a conformational alteration in the latter following receptor engagement alters or releases F allowing it to proceed towards the fusion active state and 6 HB formation, supported by studies with the MeV-H and -F glycoproteins [73,92,101].

The preponderance of data suggests that there does appear to be two major ways that paramyxoviruses have evolved to regulate F fusion activation; one represented by those employing an HN-F glycoprotein pair and the other represented by the H-F glycoprotein pair (recently reviewed in detail elsewhere [39,102,103]). However, in either case it would seem plausible that upon triggering the F glycoprotein and initiating its conformational changes leading to 6 HB formation, F would need to be free of any association with its large oligomeric partner—HN, H or G. For the henipaviruses, the findings to date suggest that HeV and NiV initiate fusion by a mechanism more in line with a clamp model. In this scenario F could be stabilized in some manner by interaction with G, perhaps maintaining F in a pre-fusion state, or it could just simply be that G and F have some propensity to specifically interact until receptor binding to G initiates some specific interaction with F triggering fusion activity and then followed by dissociation, much like a provocateur model. 

In support of a general clamp model for henipavirus fusion, it has been observed that the strength of G-F interaction is inversely proportional to fusion activity as stronger G-F interaction results in decreased fusion due to the inability of F to disassociate from G [56,89,104]. These observations are in agreement with the earlier suggestion that the henipavirus-mediated fusion mechanism is similar to MeV, which also exhibits an inverse relationship between the attachment glycoprotein (H) and F [101]. Also in accord with this fusion model are the observations that both MeV and HeV possess attachment glycoproteins that with certain mutations decrease their receptor binding activity while strengthening the interactions with their respective F glycoprotein partner [56,105]. 

Indirect support for the provocateur model also comes from data indicating that F expressed in the absence of G can be recognized by a conformation-dependent mouse mAb specific for the pre-fusion form of NiV-F [106]. These conflicting data suggest that neither a clamp model nor a provocateur model alone can fully account for all the experimental observations to date on the mechanism of henipavirus-mediated fusion. In fact, a recent report by Porotto *et al.* details a new, third fusion mechanism that is based on both the clamp and provocateur models [107]. Their new model is based on the need for continuous receptor engagement by hPIV3-HN to activate F for membrane fusion [107]. In this study, fusion intermediates were captured by using HR2 peptides localized in the target cell membrane by a cholesterol tag. It was determined that if interaction between hPIV3-HN and its receptor was interrupted during F activation and insertion into the target membrane, fusion would not occur. However, no direct HN-F interactions were assessed, such as through a co-association or a co-immunoprecipitation approach. Most recently, a further study by Porotto *et al.*, examined the hPIV3 fusion system using a bimolecular fluorescence complementation approach to follow the dynamics of the viral HN and F in living cells [108]. The authors were able to demonstrate that in this system the HN and F glycoproteins do associate prior to receptor engagement, HN drives the formation of HN and F interacting clusters at the site of membrane fusion and the interaction of the HN-F pairs of oliogmers modulate the viral glycoprotein pair’s fusogenicity [108]. This requirement of continual receptor engagement and interaction between the attachment and fusion glycoproteins was hypothesized to be applicable to all paramyxoviruses, including HeV and NiV [107]. Further research regarding this proposed mechanism in respect to HeV/NiV may be able to resolve the conflicting data supporting the two current models and clarify the mechanism of henipavirus-mediated fusion. 

## 7. Therapeutics

Licensed and efficacious antiviral therapeutics for the henipaviruses are currently not available. Ribavirin was used to treat 140 patients during the NiV outbreak in Malaysia in 1998/99, lessening the mortality rate by 35% from 54% in the control group to 32% in the treated group [109]. Without any other currently available therapeutic options, ribavirin is still considered an option for treatment, but its impact on disease progression is questionable as two HeV infected patients in 2008 showed no discernable benefit after treatment with ribavirin [8]. Additionally, chloroquine, an anti-malarial drug first demonstrated to block the proteolytic processing of HeV-F [110], was later shown to inhibit henipavirus infection *in vitro* [111]. However, treatment with chloroquine and ribavirin proved ineffective for one HeV-infected individual in 2009 as no clinical benefit was observed [112].

In subsequent animal challenge models with henipaviruses, ribavirin only delayed NiV disease and death and had no therapeutic effect against HeV infection in hamsters [113,114]. Ribavirin also only delayed HeV disease by 1 or 2 days in African green monkeys and did not prevent disease outcome [115]. Chloroquine, either alone or in combination with ribavirin, also had no therapeutic benefit in ferrets challenged with NiV or hamsters challenged with either NiV or HeV [113,116]. Thus, due to the extreme pathogenic capacity of HeV and NiV infection in people, considerable effort has been spent in developing and exploring new therapeutic options against the henipaviruses, and these treatments have primarily focused on targeting the fusion and entry step of the virus infection process and include F glycoprotein-targeted peptide fusion inhibitors and passive immunotherapy with virus neutralizing mAbs targeting the G and F glycoproteins. Here we highlight the recent developments in therapeutics that target virus entry which have shown promise in treating henipavirus infection and pathogenesis.

### 7.1. Peptide Fusion Inhibitors and Premature Fusion Triggering

Heptad peptide based fusion inhibitors corresponding to HR1 or HR2 domains of a viral fusion protein have been shown to be particularly effective at inhibiting pH-independent Class I viral fusion systems *in vitro*, such as with paramyxoviruses, when present prior to triggering the fusion process leading to the formation of the 6 HB (reviewed in [117]). Most of these approaches have utilized peptides corresponding to the HR2 domain, and they act by blocking 6 HB formation by binding in the grooves of the HR1 trimeric core and prevent the viral HR2 domains from binding. The first promising henipavirus-specific therapeutic was a 36 amino acid HR2-based fusion inhibitor (NiV-Fc2) [118] analogous to the approved HIV-1 specific therapeutic peptide enfuvirtide (Fuzeon™). Addition of exogenous NiV-Fc2 peptide could potently block HeV and NiV membrane fusion and live virus infection [118,119]. The effectiveness of the NiV-Fc2 fusion inhibitor against henipavirus infection *in vivo* in a suitable animal model is presently being evaluated [120]. 

While some approaches have focused on optimizing the length and sequence of exogenous HR2 peptides to increase their efficacy, one study determined that an HR2 peptide derived from hPIV3 proved more effective at inhibiting henipavirus mediated fusion than a peptide derived from HeV HR2 [121,122]. The increased efficacy of the hPIV3 peptide appears to be due to stronger interactions between the hPIV3 HR peptide and the binding grooves of the HR1 domains than HR peptides corresponding to the native henipavirus F HR2 peptide sequence. More recently, these observations were followed up with testing of a sequence-optimized and cholesterol tagged hPIV3-based HR2-derived peptide that targets hPIV3 and henipavirus F in the hamster-model of NiV infection [123]. Here, these cholesterol-tagged peptides could also penetrate the CNS and exhibit some effective therapeutic activity against NiV infection in the hamster model; this peptide approach could be particularly effective in treating the encephalitic manifestations of henipavirus infection. 

While there appears to be potential for peptide fusion inhibitors to be highly effective in preventing infection, there remains numerous challenges to overcome in the design and administration of such a therapeutic protocol. The peptide based fusion inhibitors should likely be small and soluble with a long half-time in the bloodstream, but they must still be able to specifically bind the heptad repeats in their target F glycoprotein in order to block 6 HB formation and membrane fusion. The time frame of effective use of peptide fusion inhibitors may also be narrow because these inhibitors are only effective if present prior to 6 HB formation but after the fusion peptide has been inserted into the target cell membrane. In addition, as with most antiviral therapeutics, peptide inhibitors would also face resistance problems since mutations may likely arise within the heptad repeat sequences of the F glycoprotein. Despite these challenges, additional *in vivo* efficacy testing of peptide fusion inhibitors of henipavirus infection merits further investigation. 

Recognizing that conformational changes occur in both G and F upon receptor binding, it was suggested earlier from mutagenesis results on HeV-G that a fusion triggered conformation could be generated in the absence of receptor, and thereby render the F-G complex ineffective (discussed above) [51]. Recently, Porotto *et al.* demonstrated that protocells expressing ephrin-B2 were capable of inactivating NiV-F and -G expressing pseudovirus by premature fusion triggering [124]. Similarly, it was demonstrated early on that soluble ephrin-B2 henipavirus receptor was capable of blocking fusion and virus entry, but its antiviral activity was presumed to be due to simply blocking virus-host cell engagement rather than also serving as a premature fusion trigger [53,54]. Not surprisingly, it was also shown that recombinant soluble versions of the ephrin-B2 receptors (EphB4 and EphB2) could also block henipavirus-mediated membrane fusion by binding to ephrin-B2 on the host cell making it unavailable for G glycoprotein binding [54]. Finally, the scenario of premature fusion triggering as a potential therapeutic approach was presented with the identification of a small molecule capable of binding hPIV3 HN and triggering F to undergo conformational change in the absence of receptor binding, again rendering this paramyxovirus glycoprotein pair ineffective and further suggesting that strategies aimed at premature fusion activation may be a viable and interesting antiviral strategy [125]. 

### 7.2. Monoclonal Antibodies

For paramyxoviruses, antibodies specific for either the F or attachment glycoproteins can neutralize virus with antibodies directed against attachment glycoproteins typically being the more predominant (reviewed in [126]). The first evidence of passive protection against a NiV challenge was demonstrated using hamsters with monospecific polyclonal antiserums against F and G [127]. Passive immune plasma therapy was also successful in the post-exposure treatment of African green monkeys infected with NiV [120]. However, the development of virus neutralizing mAbs has made passive antibody therapy development a major focus of current research. Another passive immunotherapy study in the hamster model using two murine mAbs against NiV-F and two against G was shown to completely protect the challenged animals if animals received mAbs before and immediately following challenge, and again, mAbs targeting the G glycoprotein proved more effective than mAbs targeting the F glycoprotein [128]. Similar results were also obtained in a HeV challenge model in the hamster [129].

A major advance in the development of specific mAbs has been the use of recombinant antibody technologies [130,131]. Earlier, recombinant, soluble G (sG) glycoprotein from HeV was used to isolate human mAbs. One particular human mAb (m102.4) was HeV and NiV cross-reactive and possessed extremely potent virus neutralizing activity [132,133]. *In vivo* studies have since demonstrated that m102.4 can protect animals from a lethal challenge with henipaviruses as a post-exposure application in the ferret model with NiV [134] or HeV [135]. Most recently, mAb m102.4 was tested in the African green monkey model against HeV, and again all animals could be protected from lethal disease by m102.4 when it was administered from 12 to as late as 72 hours after a lethal high dose intratracheal challenge [136]. In August 2009, m102.4 was used on a compassionate basis to save the life of a HeV-infected individual while in a coma [137]. Unfortunately, delivery and intravenous administration of only 100 mg of available antibody occurred after the onset of encephalitis and the individual died shortly thereafter. However, during the 2010 HeV emergence, prior to HeV diagnosis or the onset of clinical disease, two individuals that were considered as high risk cases of possible infection received m102.4 antibody at doses sufficient to achieve a high serum concentration, and both individuals have remained healthy [138]. Together, these findings highlight the therapeutic potential of mAb-based passive transfer modalities for treating henipavirus exposure. Presently, m102.4 is being developed further for clinical use in people. 

**Figure 3:**
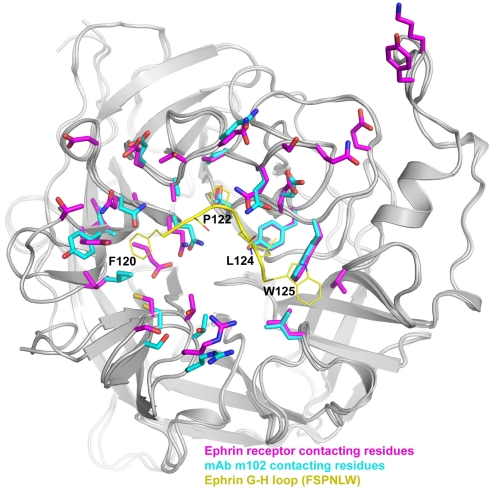
HeV-G residues that bind ephrin and monoclonal antibody m102.4. A monomeric unit of the globular head of HeV-G (gray) is shown with residues important for ephrin-B2 and -B3 binding highlighted in purple, residues required for binding mAb m102.4 shown in blue and the residues of the ephrin-B2 G-H loop in yellow. While the conformations of the residues may be slightly different, almost every residue involved in binding ephrin-B2 and -B3 is also required for binding m102.4, suggesting m102.4 prevents HeV and NiV infection by preventing ephrin-B2 and -B3 binding.

The mechanism and efficacy of m102.4-mediated neutralization is likely due to its ability to directly compete with ephrin-B2 and -B3 receptors for binding to the HeV and NiV-G glycoprotein. Indeed, this seems born out when a comparison of the G glycoprotein binding pockets used by m102.4 and ephrin-B2 and -B3 is made, which shows a remarkable series of identical contacting residues in G that are important for engaging the ephrins as well as mAb m102.4 (Figure 3) [139]. Substituting for the G-H loop of ephrin-B2 and -B3, m102.4 has a stretch of amino acid residues (L_105_APHPS_110_) that bind the henipavirus G glycoprotein with four residues considered critical for binding (L105, P107, H108 and P109). 

## 8. Concluding Remarks

The henipaviruses present credible natural and potentially deliberate biothreats to human and livestock populations. While our knowledge of the viral mechanisms involved in the infection process has increased there are still many unanswered questions. Through mutational studies it has been shown that receptor binding alone to G is not sufficient to trigger the F glycoprotein-mediated membrane fusion process. Rather, receptor engagement and membrane fusion appear to be two separate but connected steps as receptor engagement does appear to trigger conformational changes in G that likely lead to the activation of the fusion activity of F. While the crystal structures of the henipavirus G glycoproteins both alone and in complex with receptor have helped to identify the critical residues for receptor binding as well as some of the conformational changes induced by receptor binding, further studies will be required to determine the overall conformational changes that occur in the native tetrameric structure that are also postulated to lead to activation of F. 

In addition, the specific interactions between the henipavirus G and F glycoproteins, including those that are specifically required for F activation, still need to be clarified. Adding further complexity to G and F interaction and the fusion mechanism is the recently proposed model of paramyxovirus fusion that suggests fusion activation involves receptor engagement for F activation but also continual F-interaction by the receptor-bound G glycoprotein [107]. Whether the henipavirus membrane fusion process aligns more with the clamp, provocateur or continual engagement fusion model (or a combination of these) will require further clarification. Finally and perhaps most importantly, the development of human mAbs that potently inhibit the glycoprotein-mediated entry of these viruses into cells has for the first time provided a safe and effective therapeutic strategy, which is currently being used for treatment of people in emergency situations. 

## References

[1] Selvey L.A., Wells R.M., McCormack J.G., Ansford A.J., Murray K., Rogers R.J., Lavercombe P.S., Selleck P., Sheridan J.W. (1995). Infection of humans and horses by a newly described morbillivirus [see comments].. Med. J. Aust..

[2] Chua K.B., Goh K.J., Wong K.T., Kamarulzaman A., Tan P.S., Ksiazek T.G., Zaki S.R., Paul G., Lam S.K., Tan C.T. (1999). Fatal encephalitis due to Nipah virus among pig-farmers in Malaysia [see comments].. Lancet.

[3] Chua K.B., Bellini W.J., Rota P.A., Harcourt B.H., Tamin A., Lam S.K., Ksiazek T.G., Rollin P.E., Zaki S.R., Shieh W. (2000). Nipah virus: A recently emergent deadly paramyxovirus. Science.

[4] Eaton B.T., Broder C.C., Middleton D., Wang L.F. (2006). Hendra and Nipah viruses: Different and dangerous. Nat. Rev. Microbiol..

[5] Chong H.T., Kunjapan S.R., Thayaparan T., Tong J., Petharunam V., Jusoh M.R., Tan C.T. (2002). Nipah encephalitis outbreak in Malaysia, clinical features in patients from Seremban. Can. J. Neurol. Sci..

[6] Chua K.B. (2003). Nipah virus outbreak in Malaysia. J. Clin. Virol..

[7] Goh K.J., Tan C.T., Chew N.K., Tan P.S., Kamarulzaman A., Sarji S.A., Wong K.T., Abdullah B.J., Chua K.B., Lam S.K. (2000). Clinical features of Nipah virus encephalitis among pig farmers in Malaysia [see comments].. N. Engl. J. Med..

[8] Playford E.G., McCall B., Smith G., Slinko V., Allen G., Smith I., Moore F., Taylor C., Kung Y.H., Field H. (2010). Human Hendra virus encephalitis associated with equine outbreak, Australia, 2008. Emerg. Infect. Dis..

[9] Hossain M.J., Gurley E.S., Montgomery J.M., Bell M., Carroll D.S., Hsu V.P., Formenty P., Croisier A., Bertherat E., Faiz M.A. (2008). Clinical presentation of Nipah virus infection in Bangladesh. Clin. Infect. Dis..

[10] Wong K.T., Shieh W.J., Kumar S., Norain K., Abdullah W., Guarner J., Goldsmith C.S., Chua K.B., Lam S.K., Tan C.T. (2002). Nipah virus infection: Pathology and pathogenesis of an emerging paramyxoviral zoonosis. Am. J. Pathol..

[11] Chong H.T., Tan C.T. (2003). Relapsed and late-onset Nipah encephalitis, a report of three cases. Neurol. J. Southeast Asia.

[12] Tan C.T., Goh K.J., Wong K.T., Sarji S.A., Chua K.B., Chew N.K., Murugasu P., Loh Y.L., Chong H.T., Tan K.S. (2002). Relapsed and late-onset Nipah encephalitis. Ann. Neurol..

[13] Wong K.T., Robertson T., Ong B.B., Chong J.W., Yaiw K.C., Wang L.F., Ansford A.J., Tannenberg A. (2009). Human Hendra virus infection causes acute and relapsing encephalitis. Neuropathol. Appl. Neurobiol..

[14] Field H.E., Mackenzie J.S., Daszak P. (2007). Henipaviruses: Emerging paramyxoviruses associated with fruit bats. Curr. Top. Microbiol. Immunol..

[15] Gurley E.S., Montgomery J.M., Hossain M.J., Bell M., Azad A.K., Islam M.R., Molla M.A., Carroll D.S., Ksiazek T.G., Rota P.A. (2007). Person-to-person transmission of Nipah virus in a Bangladeshi community. Emerg. Infect. Dis..

[16] Homaira N., Rahman M., Hossain M.J., Epstein J.H., Sultana R., Khan M.S., Podder G., Nahar K., Ahmed B., Gurley E.S. (2010). Nipah virus outbreak with person-to-person transmission in a district of Bangladesh, 2007. Epidemiol. Infect..

[17] Luby S.P., Gurley E.S., Hossain M.J. (2009). Transmission of human infection with Nipah virus. Clin. Infect. Dis..

[18] Field H., Schaaf K., Kung N., Simon C., Waltisbuhl D., Hobert H., Moore F., Middleton D., Crook A., Smith G. (2010). Hendra virus outbreak with novel clinical features, Australia. Emerg. Infect. Dis..

[19] Bishop K.A., Broder C.C., Scheld W.M., Hammer S.M., Hughes J.M. (2008). Hendra and Nipah: Lethal Zoonotic Paramyxoviruses. Emerging Infections.

[20] (2009). Hendra Virus, Human, Equine—Australia (04): Queensland Fatal. Pro-MED-mail, Archive No. 20090903.3098. http://www.promedmail.org/.

[21] (2011). Hendra Virus, Equine—Australia (28): (Queensland, New South Wales). Pro-MED-mail, Archive No. 20111013.3061. http://www.promedmail.org/.

[22] Smith I., Broos A., de Jong C., Zeddeman A., Smith C., Smith G., Moore F., Barr J., Crameri G., Marsh G. (2011). Identifying Hendra virus diversity in pteropid bats. PLoS One.

[23] Pallister J., Middleton D., Broder C.C., Wang L.F. (2011). Henipavirus vaccine development. J. Bioterr. Biodef..

[24] Luby S.P., Hossain M.J., Gurley E.S., Ahmed B.N., Banu S., Khan S.U., Homaira N., Rota P.A., Rollin P.E., Comer J.A. (2009). Recurrent zoonotic transmission of Nipah virus into humans, Bangladesh, 2001–2007. Emerg. Infect. Dis..

[25] Stone R. (2011). Epidemiology. Breaking the chain in Bangladesh. Science.

[26] Nahar N., Sultana R., Gurley E.S., Hossain M.J., Luby S.P. (2010). Date palm sap collection: Exploring opportunities to prevent Nipah transmission. Ecohealth.

[27] Balzer M. (2011). Hendra vaccine success announced. Aust. Vet. J..

[28] Sendow I., Field H.E., Adjid A., Ratnawati A., Breed A.C., Darminto, Morrissy C., Daniels P. (2010). Screening for Nipah virus infection in West Kalimantan province, Indonesia. Zoonoses Public Health.

[29] Sendow I., Field H.E., Curran J., Darminto, Morrissy C., Meehan G., Buick T., Daniels P. (2006). Henipavirus in Pteropus vampyrus bats, Indonesia. Emerg. Infect. Dis..

[30] Wacharapluesadee S., Lumlertdacha B., Boongird K., Wanghongsa S., Chanhome L., Rollin P., Stockton P., Rupprecht C.E., Ksiazek T.G., Hemachudha T. (2005). Bat Nipah virus, Thailand. Emerg. Infect. Dis..

[31] Drexler J.F., Corman V.M., Gloza-Rausch F., Seebens A., Annan A., Ipsen A., Kruppa T., Muller M.A., Kalko E.K., Adu-Sarkodie Y. (2009). Henipavirus RNA in African bats. PLoS One.

[32] Hayman D.T., Suu-Ire R., Breed A.C., McEachern J.A., Wang L., Wood J.L., Cunningham A.A. (2008). Evidence of Henipavirus infection in West African fruit bats. PLoS One.

[33] Iehle C., Razafitrimo G., Razainirina J., Andriaholinirina N., Goodman S.M., Faure C., Georges-Courbot M.C., Rousset D., Reynes J.M. (2007). Henipavirus and Tioman virus antibodies in pteropodid bats, Madagascar. Emerg. Infect. Dis..

[34] Li Y., Wang J., Hickey A.C., Zhang Y., Wu Y., Zhang H., Yuan J., Han Z., McEachern J., Broder C.C. (2008). Antibodies to Nipah or Nipah-like viruses in bats, China. Emerg. Infect. Dis..

[35] Hayman D.T., Wang L.F., Barr J., Baker K.S., Suu-Ire R., Broder C.C., Cunningham A.A., Wood J.L. (2011). Antibodies to henipavirus or henipa-like viruses in domestic pigs in Ghana, West Africa. PLoS One.

[36] Lamb R.A., Collins P.L., Kolakofsky D., Melero J.A., Nagai Y., Oldstone M.B.A., Pringle C.R., Rima B.K., Fauquet C.M., Mayo M.A., Maniloff J., Desselberger U., Ball L.A. (2005). Family Paramyxoviridae. Virus Taxonomy: The Classification and Nomenclature of Viruses. The Eighth Report of the International Committee in Taxonomy of Viruses.

[37] Harcourt B.H., Tamin A., Ksiazek T.G., Rollin P.E., Anderson L.J., Bellini W.J., Rota P.A. (2000). Molecular characterization of Nipah virus, a newly emergent paramyxovirus. Virology.

[38] Bossart K.N., Broder C.C., Pöhlmann S., Simmons G. (2009). Paramyxovirus Entry. Viral Entry into Host Cells.

[39] Lee B., Ataman Z.A. (2011). Modes of paramyxovirus fusion: A Henipavirus perspective. Trends Microbiol..

[40] Muhlebach M.D., Mateo M., Sinn P.L., Prufer S., Uhlig K.M., Leonard V.H., Navaratnarajah C.K., Frenzke M., Wong X.X., Sawatsky B. (2011). Adherens junction protein nectin-4 is the epithelial receptor for measles virus. Nature.

[41] Noyce R.S., Bondre D.G., Ha M.N., Lin L.T., Sisson G., Tsao M.S., Richardson C.D. (2011). Tumor cell marker PVRL4 (nectin 4) is an epithelial cell receptor for measles virus. PLoS Pathog..

[42] Bowden T.A., Aricescu A.R., Gilbert R.J., Grimes J.M., Jones E.Y., Stuart D.I. (2008). Structural basis of Nipah and Hendra virus attachment to their cell-surface receptor ephrin-B2. Nat. Struct. Mol. Biol..

[43] Xu K., Rajashankar K.R., Chan Y.P., Himanen J.P., Broder C.C., Nikolov D.B. (2008). Host cell recognition by the henipaviruses: Crystal structures of the Nipah G attachment glycoprotein and its complex with ephrin-B3. Proc. Natl. Acad. Sci. U. S. A..

[44] Bowden T.A., Crispin M., Harvey D.J., Jones E.Y., Stuart D.I. (2010). Dimeric architecture of the Hendra virus attachment glycoprotein: Evidence for a conserved mode of assembly. J. Virol..

[45] Bowden T.A., Crispin M., Harvey D.J., Aricescu A.R., Grimes J.M., Jones E.Y., Stuart D.I. (2008). Crystal structure and carbohydrate analysis of Nipah virus attachment glycoprotein: A template for antiviral and vaccine design. J. Virol..

[46] Xu K., Broder C.C., Nikolov D.B. (2012).

[47] Colgrave M.L., Snelling H.J., Shiell B.J., Feng Y.R., Chan Y.P., Bossart K.N., Xu K., Nikolov D.B., Broder C.C., Michalski W.P. (2011). Site occupancy and glycan compositional analysis of two soluble recombinant forms of the attachment glycoprotein of Hendra virus. Glycobiology.

[48] Bossart K.N., Crameri G., Dimitrov A.S., Mungall B.A., Feng Y.R., Patch J.R., Choudhary A., Wang L.F., Eaton B.T., Broder C.C. (2005). Receptor binding, fusion inhibition, and induction of cross-reactive neutralizing antibodies by a soluble G glycoprotein of Hendra virus. J. Virol..

[49] Yuan P., Swanson K.A., Leser G.P., Paterson R.G., Lamb R.A., Jardetzky T.S. (2011). Structure of the Newcastle disease virus hemagglutinin-neuraminidase (HN) ectodomain reveals a four-helix bundle stalk. Proc. Natl. Acad. Sci. U. S. A..

[50] Kelley L.A., Sternberg M.J. (2009). Protein structure prediction on the Web: A case study using the Phyre server. Nat. Protoc..

[51] Bishop K.A., Hickey A.C., Khetawat D., Patch J.R., Bossart K.N., Zhu Z., Wang L.F., Dimitrov D.S., Broder C.C. (2008). Residues in the stalk domain of the Hendra virus G glycoprotein modulate conformational changes associated with receptor binding. J. Virol..

[52] Yuan P., Thompson T.B., Wurzburg B.A., Paterson R.G., Lamb R.A., Jardetzky T.S. (2005). Structural studies of the parainfluenza virus 5 hemagglutinin-neuraminidase tetramer in complex with its receptor, sialyllactose. Structure.

[53] Bonaparte M.I., Dimitrov A.S., Bossart K.N., Crameri G., Mungall B.A., Bishop K.A., Choudhry V., Dimitrov D.S., Wang L.F., Eaton B.T. (2005). Ephrin-B2 ligand is a functional receptor for Hendra virus and Nipah virus. Proc. Natl. Acad. Sci. U. S. A..

[54] Negrete O.A., Levroney E.L., Aguilar H.C., Bertolotti-Ciarlet A., Nazarian R., Tajyar S., Lee B. (2005). EphrinB2 is the entry receptor for Nipah virus, an emergent deadly paramyxovirus. Nature.

[55] Negrete O.A., Wolf M.C., Aguilar H.C., Enterlein S., Wang W., Muhlberger E., Su S.V., Bertolotti-Ciarlet A., Flick R., Lee B. (2006). Two key residues in ephrinB3 are critical for its use as an alternative receptor for Nipah virus. PLoS Pathog..

[56] Bishop K.A., Stantchev T.S., Hickey A.C., Khetawat D., Bossart K.N., Krasnoperov V., Gill P., Feng Y.R., Wang L., Eaton B.T. (2007). Identification of Hendra virus G glycoprotein residues that are critical for receptor binding. J. Virol..

[57] Pasquale E.B. (2010). Eph receptors and ephrins in cancer: Bidirectional signalling and beyond. Nat. Rev. Cancer.

[58] Lackmann M., Boyd A.W. (2008). Eph, a protein family coming of age: More confusion, insight, or complexity?. Sci. Signal..

[59] Bossart K.N., Tachedjian M., McEachern J.A., Crameri G., Zhu Z., Dimitrov D.S., Broder C.C., Wang L.F. (2008). Functional studies of host-specific ephrin-B ligands as Henipavirus receptors. Virology.

[60] Gale N.W., Baluk P., Pan L., Kwan M., Holash J., DeChiara T.M., McDonald D.M., Yancopoulos G.D. (2001). Ephrin-B2 selectively marks arterial vessels and neovascularization sites in the adult, with expression in both endothelial and smooth-muscle cells. Dev. Biol..

[61] Poliakov A., Cotrina M., Wilkinson D.G. (2004). Diverse roles of eph receptors and ephrins in the regulation of cell migration and tissue assembly. Dev. Cell.

[62] Pasquale E.B. (2008). Eph-ephrin bidirectional signaling in physiology and disease. Cell.

[63] Hooper P., Zaki S., Daniels P., Middleton D. (2001). Comparative pathology of the diseases caused by Hendra and Nipah viruses. Microbes Infect..

[64] Wong K.T. (2010). Emerging epidemic viral encephalitides with a special focus on henipaviruses. Acta Neuropathol..

[65] Bossart K.N., McEachern J.A., Hickey A.C., Choudhry V., Dimitrov D.S., Eaton B.T., Wang L.F. (2007). Neutralization assays for differential Henipavirus serology using Bio-Plex Protein Array Systems. J. Virol. Methods.

[66] Negrete O.A., Chu D., Aguilar H.C., Lee B. (2007). Single amino acid changes in the Nipah and Hendra virus attachment glycoproteins distinguish ephrinB2 from ephrinB3 Usage. J. Virol..

[67] Toth J., Cutforth T., Gelinas A.D., Bethoney K.A., Bard J., Harrison C.J. (2001). Crystal structure of an ephrin ectodomain. Dev. Cell.

[68] Koolpe M., Burgess R., Dail M., Pasquale E.B. (2005). EphB receptor-binding peptides identified by phage display enable design of an antagonist with ephrin-like affinity. J. Biol. Chem..

[69] Nikolov D.B., Li C., Barton W.A., Himanen J.P. (2005). Crystal structure of the ephrin-B1 ectodomain: Implications for receptor recognition and signaling. Biochemistry.

[70] Navaratnarajah C.K., Oezguen N., Rupp L., Kay L., Leonard V.H., Braun W., Cattaneo R. (2011). The heads of the measles virus attachment protein move to transmit the fusion-triggering signal. Nat. Struct. Mol. Biol..

[71] Guillaume V., Aslan H., Ainouze M., Guerbois M., Wild T.F., Buckland R., Langedijk J.P. (2006). Evidence of a potential receptor-binding site on the Nipah virus G protein (NiV-G): Identification of globular head residues with a role in fusion promotion and their localization on an NiV-G structural model. J. Virol..

[72] Yuan J., Marsh G., Khetawat D., Broder C.C., Wang L.F., Shi Z. (2011). Mutations in the G-H loop region of ephrin-B2 can enhance Nipah virus binding and infection. J. Gen. Virol..

[73] Corey E.A., Iorio R.M. (2007). Mutations in the stalk of the measles virus hemagglutinin protein decrease fusion but do not interfere with virus-specific interaction with the homologous fusion protein. J. Virol..

[74] Brindley M.A., Plemper R.K. (2010). Blue native PAGE and biomolecular complementation reveal a tetrameric or higher-order oligomer organization of the physiological measles virus attachment protein H. J. Virol..

[75] Pager C.T., Dutch R.E. (2005). Cathepsin L is involved in proteolytic processing of the Hendra virus fusion protein. J. Virol..

[76] Meulendyke K.A., Wurth M.A., McCann R.O., Dutch R.E. (2005). Endocytosis plays a critical role in proteolytic processing of the Hendra virus fusion protein. J. Virol..

[77] Vogt C., Eickmann M., Diederich S., Moll M., Maisner A. (2005). Endocytosis of the Nipah virus glycoproteins. J. Virol..

[78] Moll M., Diederich S., Klenk H.D., Czub M., Maisner A. (2004). Ubiquitous activation of the Nipah virus fusion protein does not require a basic amino Acid at the cleavage site. J. Virol..

[79] Lou Z., Xu Y., Xiang K., Su N., Qin L., Li X., Gao G.F., Bartlam M., Rao Z. (2006). Crystal structures of Nipah and Hendra virus fusion core proteins. FEBS J..

[80] Popa A., Pager C.T., Dutch R.E. (2011). C-terminal tyrosine residues modulate the fusion activity of the Hendra virus fusion protein. Biochemistry.

[81] Weise C., Erbar S., Lamp B., Vogt C., Diederich S., Maisner A. (2010). Tyrosine residues in the cytoplasmic domains affect sorting and fusion activity of the Nipah virus glycoproteins in polarized epithelial cells. J. Virol..

[82] Yin H.S., Wen X., Paterson R.G., Lamb R.A., Jardetzky T.S. (2006). Structure of the parainfluenza virus 5 F protein in its metastable, prefusion conformation. Nature.

[83] Yin H.S., Paterson R.G., Wen X., Lamb R.A., Jardetzky T.S. (2005). Structure of the uncleaved ectodomain of the paramyxovirus (hPIV3) fusion protein. Proc. Natl. Acad. Sci. U. S. A..

[84] Lamb R.A., Jardetzky T.S. (2007). Structural basis of viral invasion: Lessons from paramyxovirus F. Curr. Opin. Struct. Biol..

[85] Smith E.C., Popa A., Chang A., Masante C., Dutch R.E. (2009). Viral entry mechanisms: The increasing diversity of paramyxovirus entry. FEBS J..

[86] Karron R.A., Buonagurio D.A., Georgiu A.F., Whitehead S.S., Adamus J.E., Clements-Mann M.L., Harris D.O., Randolph V.B., Udem S.A., Murphy B.R. (1997). Respiratory syncytial virus (RSV) SH and G proteins are not essential for viral replication *in vitro*: Clinical evaluation and molecular characterization of a cold-passaged, attenuated RSV subgroup B mutant. Proc. Natl. Acad. Sci. U. S. A..

[87] Techaarpornkul S., Barretto N., Peeples M.E. (2001). Functional analysis of recombinant respiratory syncytial virus deletion mutants lacking the small hydrophobic and/or attachment glycoprotein gene. J. Virol..

[88] Dutch R.E., Joshi S.B., Lamb R.A. (1998). Membrane fusion promoted by increasing surface densities of the paramyxovirus F and HN proteins: Comparison of fusion reactions mediated by simian virus 5 F, human parainfluenza virus type 3 F, and influenza virus HA. J. Virol..

[89] Aguilar H.C., Matreyek K.A., Choi D.Y., Filone C.M., Young S., Lee B. (2007). Polybasic KKR motif in the cytoplasmic tail of Nipah virus fusion protein modulates membrane fusion by inside-out signaling. J. Virol..

[90] Aguilar H.C., Ataman Z.A., Aspericueta V., Fang A.Q., Stroud M., Negrete O.A., Kammerer R.A., Lee B. (2009). A novel receptor-induced activation site in the Nipah virus attachment glycoprotein (G) involved in triggering the fusion glycoprotein (F).. J. Biol. Chem..

[91] Whitman S.D., Smith E.C., Dutch R.E. (2009). Differential rates of protein folding and cellular trafficking for the Hendra virus F and G proteins: Implications for F-G complex formation. J. Virol..

[92] Plemper R.K., Hammond A.L., Cattaneo R. (2001). Measles virus envelope glycoproteins hetero-oligomerize in the endoplasmic reticulum. J. Biol. Chem..

[93] Stone-Hulslander J., Morrison T.G. (1997). Detection of an interaction between the HN and F proteins in Newcastle disease virus-infected cells. J. Virol..

[94] Tong S., Compans R.W. (1999). Alternative mechanisms of interaction between homotypic and heterotypic parainfluenza virus HN and F proteins. J. Gen. Virol..

[95] Paterson R.G., Johnson M.L., Lamb R.A. (1997). Paramyxovirus fusion (F) protein and hemagglutinin-neuraminidase (HN) protein interactions: Intracellular retention of F and HN does not affect transport of the homotypic HN or F protein. Virology.

[96] Whitman S.D., Dutch R.E. (2007). Surface density of the Hendra G protein modulates Hendra F protein-promoted membrane fusion: Role for Hendra G protein trafficking and degradation. Virology.

[97] Sergel T., McGinnes L.W., Peeples M.E., Morrison T.G. (1993). The attachment function of the Newcastle disease virus hemagglutinin-neuraminidase protein can be separated from fusion promotion by mutation. Virology.

[98] Melanson V.R., Iorio R.M. (2004). Amino acid substitutions in the F-specific domain in the stalk of the newcastle disease virus HN protein modulate fusion and interfere with its interaction with the F protein. J. Virol..

[99] Li J., Quinlan E., Mirza A., Iorio R.M. (2004). Mutated form of the Newcastle disease virus hemagglutinin-neuraminidase interacts with the homologous fusion protein despite deficiencies in both receptor recognition and fusion promotion. J. Virol..

[100] Connolly S.A., Leser G.P., Jardetzky T.S., Lamb R.A. (2009). Bimolecular complementation of paramyxovirus fusion and hemagglutinin-neuraminidase proteins enhances fusion: Implications for the mechanism of fusion triggering. J. Virol..

[101] Plemper R.K., Hammond A.L., Gerlier D., Fielding A.K., Cattaneo R. (2002). Strength of envelope protein interaction modulates cytopathicity of measles virus. J. Virol..

[102] Plemper R.K., Brindley M.A., Iorio R.M. (2011). Structural and mechanistic studies of measles virus illuminate paramyxovirus entry. PLoS Pathog..

[103] Dutch R.E. (2010). Entry and fusion of emerging paramyxoviruses. PLoS Pathog..

[104] Aguilar H.C., Matreyek K.A., Filone C.M., Hashimi S.T., Levroney E.L., Negrete O.A., Bertolotti-Ciarlet A., Choi D.Y., McHardy I., Fulcher J.A. (2006). N-Glycans on Nipah virus fusion protein protect against neutralization but reduce membrane fusion and viral entry. J. Virol..

[105] Corey E.A., Iorio R.M. (2009). Measles virus attachment proteins with impaired ability to bind CD46 interact more efficiently with the homologous fusion protein. Virology.

[106] Chan Y.P., Broder C.C. (2012).

[107] Porotto M., Devito I., Palmer S.G., Jurgens E.M., Yee J.L., Yokoyama C.C., Pessi A., Moscona A. (2011). Spring-loaded model revisited: Paramyxovirus fusion requires engagement of a receptor binding protein beyond initial triggering of the fusion protein. J. Virol..

[108] Porotto M., Palmer S.G., Palermo L.M., Moscona A. (2011). Mechanism of fusion triggering by human parainfluenza virus type III: Communication between viral glycoproteins during entry. J. Biol. Chem..

[109] Chong H.T., Kamarulzaman A., Tan C.T., Goh K.J., Thayaparan T., Kunjapan S.R., Chew N.K., Chua K.B., Lam S.K. (2001). Treatment of acute Nipah encephalitis with ribavirin. Ann. Neurol..

[110] Pager C.T., Wurth M.A., Dutch R.E. (2004). Subcellular localization and calcium and pH requirements for proteolytic processing of the Hendra virus fusion protein. J. Virol..

[111] Porotto M., Orefice G., Yokoyama C.C., Mungall B.A., Realubit R., Sganga M.L., Aljofan M., Whitt M., Glickman F., Moscona A. (2009). Simulating henipavirus multicycle replication in a screening assay leads to identification of a promising candidate for therapy. J. Virol..

[112] (2009). Hendra Virus, Human, Equine—Australia (05): Queensland. Pro-MED-mail, Archive No. 20090910.3189. http://www.promedmail.org/.

[113] Freiberg A.N., Worthy M.N., Lee B., Holbrook M.R. (2010). Combined chloroquine and ribavirin treatment does not prevent death in a hamster model of Nipah and Hendra virus infection. J. Gen. Virol..

[114] Georges-Courbot M.C., Contamin H., Faure C., Loth P., Baize S., Leyssen P., Neyts J., Deubel V. (2006). Poly(I)-poly(C12U) but not ribavirin prevents death in a hamster model of Nipah virus infection. Antimicrob. Agents Chemother..

[115] Rockx B., Bossart K.N., Feldmann F., Geisbert J.B., Hickey A.C., Brining D., Callison J., Safronetz D., Marzi A., Kercher L. (2010). A novel model of lethal Hendra virus infection in African green monkeys and the effectiveness of ribavirin treatment. J. Virol..

[116] Pallister J., Middleton D., Crameri G., Yamada M., Klein R., Hancock T.J., Foord A., Shiell B., Michalski W., Broder C.C. (2009). Chloroquine administration does not prevent Nipah virus infection and disease in ferrets. J. Virol..

[117] Bossart K.N., Broder C.C. (2006). Developments towards effective treatments for Nipah and Hendra virus infection. Expert Rev. Anti Infect. Ther..

[118] Bossart K.N., Wang L.F., Eaton B.T., Broder C.C. (2001). Functional expression and membrane fusion tropism of the envelope glycoproteins of Hendra virus. Virology.

[119] Bossart K.N., Mungall B.A., Crameri G., Wang L.F., Eaton B.T., Broder C.C. (2005). Inhibition of Henipavirus fusion and infection by heptad-derived peptides of the Nipah virus fusion glycoprotein. Virol. J..

[120] Geisbert T.W., Broder C.C. (2012).

[121] Porotto M., Carta P., Deng Y., Kellogg G.E., Whitt M., Lu M., Mungall B.A., Moscona A. (2007). Molecular determinants of antiviral potency of paramyxovirus entry inhibitors. J. Virol..

[122] Porotto M., Doctor L., Carta P., Fornabaio M., Greengard O., Kellogg G.E., Moscona A. (2006). Inhibition of Hendra virus fusion. J. Virol..

[123] Porotto M., Rockx B., Yokoyama C.C., Talekar A., Devito I., Palermo L.M., Liu J., Cortese R., Lu M., Feldmann H. (2010). Inhibition of Nipah virus infection *in vivo*: Targeting an early stage of paramyxovirus fusion activation during viral entry. PLoS Pathog..

[124] Porotto M., Yi F., Moscona A., LaVan D.A. (2011). Synthetic protocells interact with viral nanomachinery and inactivate pathogenic human virus. PLoS One.

[125] Farzan S.F., Palermo L.M., Yokoyama C.C., Orefice G., Fornabaio M., Sarkar A., Kellogg G.E., Greengard O., Porotto M., Moscona A. (2011). Premature activation of the paramyxovirus fusion protein before target cell attachment with corruption of the viral fusion machinery. J. Biol. Chem..

[126] Broder C.C., Levine M.M., Dougan G., Good M.F., Liu M.A., Nabel G.J., Nataro J.P., Rappuoli R. (2010). Therapeutics and Vaccines against Hendra and Nipah Viruses. New Generation Vaccines.

[127] Guillaume V., Contamin H., Loth P., Georges-Courbot M.C., Lefeuvre A., Marianneau P., Chua K.B., Lam S.K., Buckland R., Deubel V. (2004). Nipah virus: Vaccination and passive protection studies in a hamster model. J. Virol..

[128] Guillaume V., Contamin H., Loth P., Grosjean I., Courbot M.C., Deubel V., Buckland R., Wild T.F. (2006). Antibody prophylaxis and therapy against Nipah virus infection in hamsters. J. Virol..

[129] Guillaume V., Wong K.T., Looi R.Y., Georges-Courbot M.C., Barrot L., Buckland R., Wild T.F., Horvat B. (2009). Acute Hendra virus infection: Analysis of the pathogenesis and passive antibody protection in the hamster model. Virology.

[130] Hayden M.S., Gilliland L.K., Ledbetter J.A. (1997). Antibody engineering. Curr. Opin. Immunol..

[131] Rader C., Barbas C.F. (1997). Phage display of combinatorial antibody libraries. Curr. Opin. Biotechnol..

[132] Zhu Z., Bossart K.N., Bishop K.A., Crameri G., Dimitrov A.S., McEachern J.A., Feng Y., Middleton D., Wang L.F., Broder C.C. (2008). Exceptionally potent cross-reactive neutralization of Nipah and Hendra viruses by a human monoclonal antibody. J. Infect. Dis..

[133] Zhu Z., Dimitrov A.S., Bossart K.N., Crameri G., Bishop K.A., Choudhry V., Mungall B.A., Feng Y.R., Choudhary A., Zhang M.Y. (2006). Potent neutralization of Hendra and Nipah viruses by human monoclonal antibodies. J. Virol..

[134] Bossart K.N., Zhu Z., Middleton D., Klippel J., Crameri G., Bingham J., McEachern J.A., Green D., Hancock T.J., Chan Y.P. (2009). A neutralizing human monoclonal antibody protects against lethal disease in a new ferret model of acute Nipah virus infection. PLoS Pathog..

[135] Pallister J., Middleton D., Broder C.C. (2012).

[136] Bossart K.N., Geisbert T.W., Feldmann H., Zhu Z., Feldmann F., Geisbert J.B., Yan L., Feng Y.R., Brining D., Scott D. (2011). A neutralizing human monoclonal antibody protects African green monkeys from Hendra virus challenge. Sci. Transl. Med..

[137] Playford G. (2009). Pathology Queensland, Herston, Queensland, Austral. Personal Communication..

[138] (2010). Hendra Virus, Equine—Australia (05): (Queensland) Human Exposure. Pro-MED-mail, Archive No. 20100527.1761. http://www.promedmail.org/.

[139] Xu K., Broder C.C., Nikolov D.B. (2012).

